# PET/CT scanning with 3D acquisition is feasible for quantifying myocardial blood flow when diagnosing coronary artery disease

**DOI:** 10.1186/s13550-017-0296-x

**Published:** 2017-06-05

**Authors:** Osamu Manabe, Masanao Naya, Tadao Aikawa, Masahiko Obara, Keiichi Magota, Markus Kroenke, Noriko Oyama-Manabe, Kenji Hirata, Daiki Shinyama, Chietsugu Katoh, Nagara Tamaki

**Affiliations:** 1 Department of Nuclear Medicine, Hokkaido University Graduate School of Medicine, Sapporo, Japan; 2 Department of Cardiovascular Medicine, Hokkaido University Graduate School of Medicine, Kita-15, Nishi-7, Kita-ku, Sapporo, 060-8638 Japan; 3 Department of Nuclear Medicine, Klinikum rechts der Isar, Technical University of Munich, Munich, Germany; 4 Department of Diagnostic and Interventional Radiology, Hokkaido University Hospital, Sapporo, Japan; 5 Philips Electronics Japan, Tokyo, Japan; 6 Faculty of Health Sciences, Hokkaido University Graduate School of Medicine, Sapporo, Japan

**Keywords:** Myocardial blood flow, Coronary flow reserve, Positron emission tomography, ^15^O-water, Three-dimensional data acquisition

## Abstract

**Background:**

The quantification of myocardial blood flow (MBF) and coronary flow reserve (CFR) are useful approaches for evaluating the functional severity of coronary artery disease (CAD). ^15^O-water positron emission tomography (PET) is considered the gold standard method for MBF quantification. However, MBF measurements in ^15^O-water PET with three-dimensional (3D) data acquisition, attenuation correction using computed tomography (CT), and time of flight have not been investigated in detail or validated. We conducted this study to evaluate the diagnostic potential of MBF measurements using PET/CT for a comparison of a control group and patients suspected of having CAD.

**Results:**

Twenty-four patients with known or suspected CAD and eight age-matched healthy volunteers underwent rest and pharmacological stress perfusion studies with ^15^O-water PET/CT. The whole and three regional (left anterior descending (LAD), left circumflex (LCX), and right coronary artery (RCA) territory) MBF values were estimated. The CFR was computed as the ratio of the MBF during adenosine triphosphate-induced stress to the MBF at rest. The inter-observer variability was assessed by two independent observers. PET/CT using a ^15^O-water dose of 500 MBq and 3D data acquisition showed good image quality. A strong inter-observer correlation was detected in both the whole MBF analysis and the regional analysis with high intra-class correlation coefficients (*r* > 0.90, *p* < 0.001). Regional MBF at rest (LAD, 0.82 ± 0.15 ml/min/g; LCX, 0.83 ± 0.17 ml/min/g; RCA, 0.71 ± 0.20 ml/min/g; *p* = 0.74), MBF at stress (LAD, 3.77 ± 1.00 ml/min/g; LCX, 3.56 ± 1.01 ml/min/g; RCA, 3.27 ± 1.04 ml/min/g; *p* = 0.62), and CFR (LAD, 4.64 ± 0.90; LCX, 4.30 ± 0.64; RCA, 4.64 ± 0.96; *p* = 0.66) of the healthy volunteers showed no significant difference among the three regions. The global CFR of the patients was significantly lower than that of the volunteers (2.75 ± 0.81 vs. 4.54 ± 0.66, *p* = 0.0002). The regional analysis of the patients demonstrated that the CFR tended to be lower in the stenotic region compared to the non-stenotic region (2.43 ± 0.81 vs. 2.95 ± 0.92, *p* = 0.052).

**Conclusions:**

^15^O-water PET/CT with 3D data acquisition can be reliably used for the quantification of functional MBF and CFR in CAD patients.

## Background

The quantifications of myocardial blood flow (MBF) and coronary flow reserve (CFR) are useful approaches for evaluating the functional severity of coronary artery disease (CAD) [1, 2]. ^15^O-water positron emission tomography (PET) is considered the gold standard method for the quantification of MBF, because it is the only method using a freely diffusible tracer with a 100% extraction fraction [3, 4]. However, MBF quantification using PET/computed tomography (CT) with three-dimensional (3D) data acquisition presents technical challenges due to increased scattering. Accordingly, PET/CT MBF measurements using ^15^O-water should be validated by comparing CAD patients and an age-matched healthy control group. The aim of this study was to evaluate the diagnostic potential of MBF and CFR measurements using ^15^O-water PET/CT with 3D data acquisition.

## Methods

### Study subjects

Twenty-four patients with CAD or suspected CAD (age 62 ± 12 years; 20 males, four females) and eight age-matched healthy volunteers (age 58 ± 6 years; seven males, one female) underwent ^15^O-water PET/CT. All patients underwent coronary angiography with standard techniques; a ≥50% dia stenosis was considered significant. This prospective study was approved by the Ethics Committee of Hokkaido University Hospital (UMIN ID, UMIN000013003). Written informed consent was obtained from all subjects.

### PET imaging acquisition

PET was performed using a Gemini TF PET/CT scanner (Philips Healthcare, Cleveland, OH) with a 64-row-detector CT system. The participants were instructed to fast for at least 4 h and to abstain from caffeine-containing products for at least 24 h prior to the PET/CT scan [5]. A prospective ECG-triggered CT for calculating the Agatston score was performed with following parameters: tube voltage, 120 kVp; effective tube current, 100 mAs; rotation time, 0.4 s; collimation width, 40 × 0.625 mm; and slice thickness, 2.5 mm. After a low-dose CT scan at free breathing for attenuation and scatter correction, 500 MBq of ^15^O-water was slowly administered intravenously (100 s) with a simultaneous 6-min list-mode acquisition. All PET scans were executed in 3D mode.

Pharmacological stress was induced by an intravenous injection of adenosine triphosphate (ATP) (160 μg/kg/min) at 3 min before the emission scanning. The subject’s heart rate and blood pressure were recorded before and at 1-min intervals during the ATP infusion. Before the attenuation correction, manual registration was created in coronal, sagittal, and transaxial views. The rest and stress PET/CT images were visually aligned for proper registration, carefully ensuring that the left ventricular myocardial activity on PET did not overlap with the lung parenchyma on CT [6]. The list-mode data were subdivided into 24 serial frames (18 × 10 and 6 × 30 s).

Attenuation-corrected radioactivity images were reconstructed using a 3D-row action maximum-likelihood algorithm (iterations, 2; relaxation parameter, 0.012). The calcium-scoring CT was reconstructed using a window centered at 75% of the RR interval and transferred to commercially available software (Intelli Space Portal ver. 5; Philips Electronics Japan, Tokyo) to calculate the Agatston score. A calcified lesion was defined as an area of at least three connected pixels with an attenuation of >130 Hounsfield units (HU). The Agatston score was calculated by multiplying the area of each lesion with a weighted attenuation score dependent on the maximal attenuation within the lesion (score of 1 for 130–199 HU, 2 for 200–299 HU, 3 for 300–399 HU, and 4 for ≥400 HU) [7]. The total radiation dose was estimated about 4.2 mSv (less than 0.1 mSv for the scout, 1.2 mSv for the Agatston score, 0.7 mSv for the attenuation correction CT, and 1.1 mSv for each ^15^O-water PET scan).

### Quantification of MBF

The MBF quantification was achieved using a software program developed in-house that semi-automatically defines regions of interest (ROI) for the left ventricular (LV) blood pool and the LV myocardium. The MBF was calculated using a one-tissue-compartment tracer kinetic model, including a myocardium-to-blood spillover correction [8]. The CFR was calculated as the ratio of the MBF during stress to the MBF at rest [9].

The MBF and CFR values were calculated from the global LV and the three coronary regions [10]. We first assessed the inter-observer variability of MBF and CFR by two independent observers (authors M.N. and T.A.). Each observer put ROIs over the whole LV myocardium and within the LV cavity to estimate the MBF and CFR using the same dynamic data. The variability of MBF and CFR compared to the healthy control group was then evaluated. We compared the MBF and CFR values from the global LV between the healthy volunteers and the patients. Finally, the regional CFR was compared among healthy volunteers and the region with and without significant stenosis in the patients who had not received prior percutaneous coronary intervention (PCI) or coronary artery bypass grafting (CABG).

### Statistical analysis

The data are expressed as mean ± standard deviation (SD). The Wilcoxon rank-sum test was used for continuous variables. Pearson’s correlation coefficient was used to evaluate the concordance between the MBF and CFR values. An analysis of variance (ANOVA) was used to compare regional CFRs among the three coronary territories. The correlation between the MBF and CFR values was assessed using linear regression analyses and intra-class correlation coefficients (ICCs). For each analysis, *p* values <0.05 were considered significant. JMP ver. 12 software (SAS Institute, Cary, NC) was used for the data analyses.

## Results

### Subjects’ backgrounds

The subjects’ backgrounds are summarized in Table 1. There was no significant difference in the age, body mass index (BMI), gender, or incidence of dyslipidemia between the volunteers and patients. However, the frequencies of hypertension, diabetes, and smoking were significantly higher in the patients compared to the volunteers. Nine patients (37.5%) had a history of PCI or CABG therapy before the PET/CT scan. The hemodynamic data included the heart rate (HR), systolic blood pressure (SBP), diastolic blood pressure (DBP), and rate pressure product (RPP). The HR and RPP in all subjects were significantly higher at stress compared to the rest scan (Table 2).

**Table 1:** The subjects’ backgrounds

	Volunteers (*n* = 8)	Patients (*n* = 24)	*p* value
Age (years)	58 ± 6	62 ± 12	0.10
Gender (male)	7 (88%)	20 (83%)	0.78
BMI (kg/m^2^)	24 ± 3	25 ± 3	0.43
Hypertension	1 (13%)	8 (33%)	0.0078
Diabetes mellitus	0 (0%)	9 (38%)	0.041
Dyslipidemia	5 (63%)	8 (33%)	0.83
Smoking	0 (0%)	11 (46%)	0.018
Prior PCI or CABG	0 (0%)	9 (38%)	0.041
Agatston score	38 ± 48	911 ± 1,000	0.0024

*BMI* body mass index, *PCI* percutaneous coronary intervention, *CABG* coronary artery bypass graft

**Table 2:** Hemodynamic data

		Volunteers	Patients	*p* value
Rest	HR (bpm)	59 ± 5	62 ± 9	0.24
	SBP (mmHg)	109 ± 11	119 ± 15	0.074
	DBP (mmHg)	63 ± 9	62 ± 9	0.86
	RPP (bpm mmHg)	6486 ± 1,029	7520 ± 1,471	0.050
Stress	HR (bpm)	79 ± 9*	77 ± 11*	0.63
	SBP (mmHg)	102 ± 11	106 ± 16	0.35
	DBP (mmHg)	59 ± 9	56 ± 12	0.45
	RPP (bpm mmHg)	8069 ± 1345*	8225 ± 1968*	0.66

*HR* heart rate, *bpm* beat per minute, *SBP* systolic blood pressure, *DBP* diastolic blood pressure, *RPP* rate pressure product

*p < 0.01 compared to at rest

### Inter-observer correlation of MBFs and CFRs

**Fig. 1:**
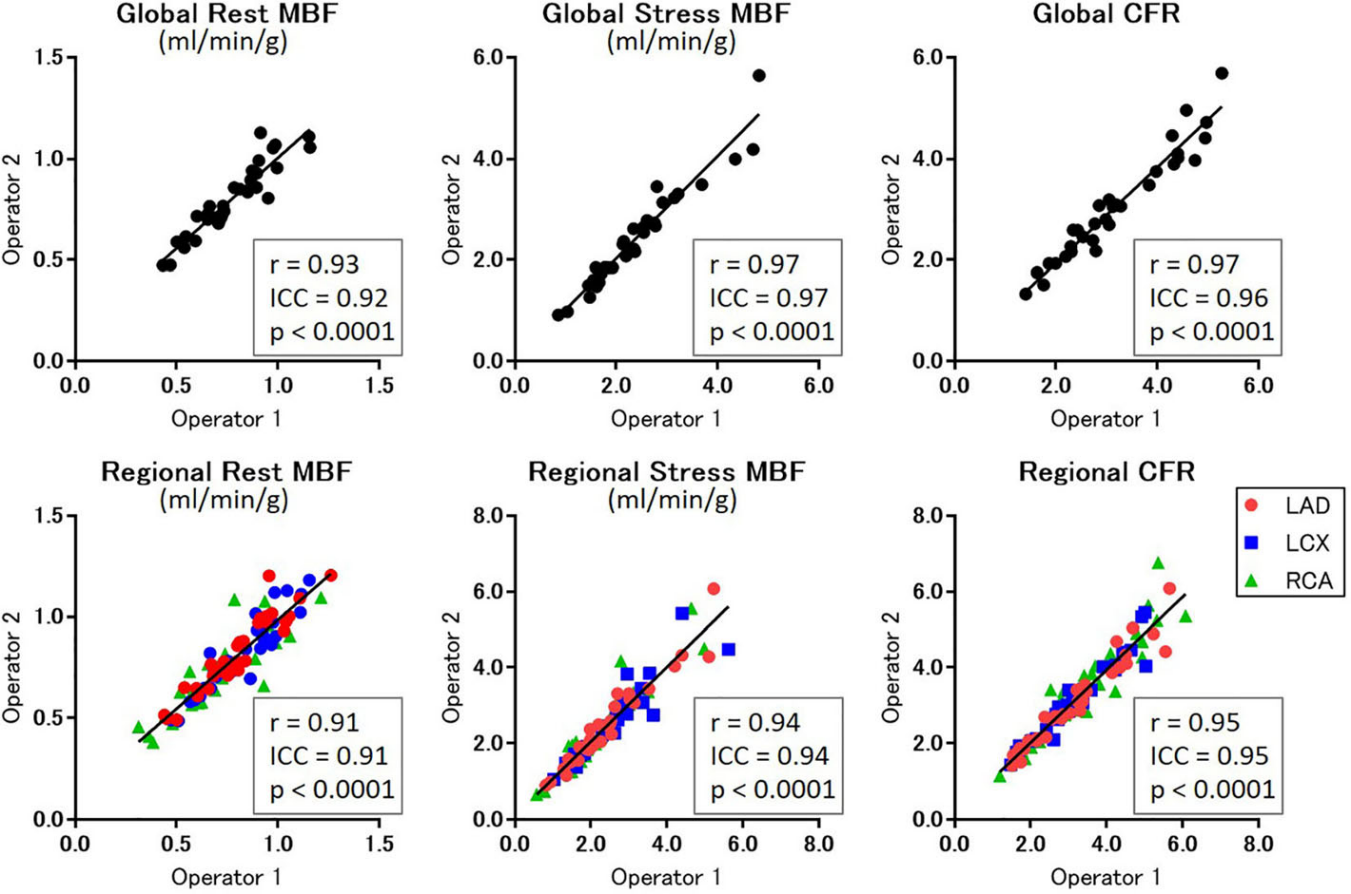
Inter-observer repeatability of MBF and CFR. The results of the linear regression analysis of the variability in MBF and CFR between the two independent observers are shown. Strong inter-observer correlations were observed in both the global and regional analyses

The variability in MBF and CFR values between the two independent observers is illustrated in Fig. 1. A strong inter-observer correlation was found for both the global analysis (*r* = 0.91 for the rest MBF, *r* = 0.94 for the stress MBF, and *r* = 0.95 for the CFR) and the regional analysis (*r* = 0.93 for the rest MBF, *r* = 0.97 for the stress MBF, and *r* = 0.97 for the CFR).

### The variability of MBF and CFR in the control group

The estimated regional MBF at rest, MBF at stress, and CFR of the healthy volunteers showed no significant difference among the three coronary regions (Table 3).

**Table 3:** Regional myocardial blood flow and coronary flow reserve of volunteers

	LAD territory	LCX territory	RCA territory	*p* value
Rest MBF (ml/min/g)	0.82 ± 0.15	0.83 ± 0.17	0.71 ± 0.20	0.74
Stress MBF (ml/min/g)	3.77 ± 1.00	3.56 ± 1.01	3.27 ± 1.04	0.62
CFR	4.64 ± 0.90	4.30 ± 0.64	4.64 ± 0.96	0.66

*LAD* left anterior descending, *LCX* left circumflex, *RCA* right coronary artery, *MBF* myocardial blood flow, *CFR* coronary flow reserve

### Comparison between healthy volunteers and CAD patients

**Fig. 2:**
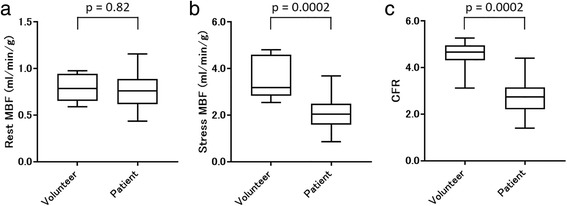
Comparison of global left ventricle MBF and CFR between the volunteers and patients. Rest MBF showed no significant difference between the volunteers and patients (**a**). However, the stress MBF (**b** 2.04 ± 0.62 vs 3.56 ± 0.91 ml/min/g, *p* = 0.0002) and whole CFR (**c** 2.75 ± 0.81 vs 4.54 ± 0.66, *p* = 0.0002) of the volunteers were significantly higher than those of the patients

**Fig. 3:**
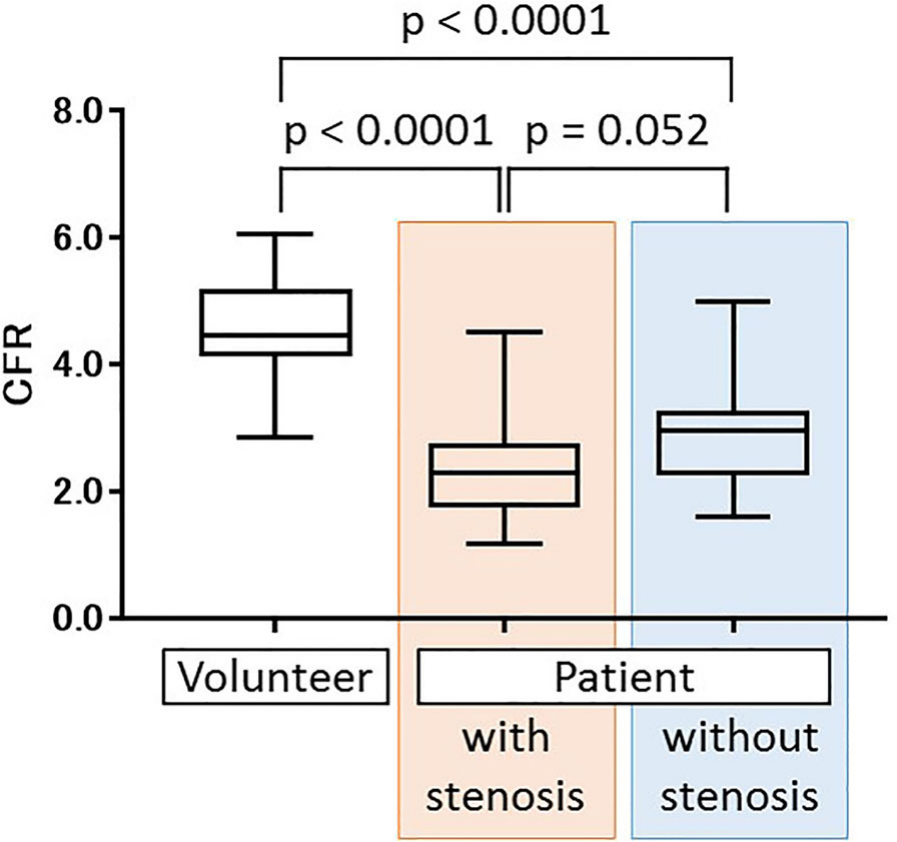
The regional CFR values of the healthy volunteers were significantly higher than those of the patients (4.53 ± 0.82 vs 2.63 ± 0.88, *p* < 0.0001). Among the patients, the CFR values of the stenotic regions tended to be lower compared to those of the non-stenotic regions (2.43 ± 0.81 vs 2.95 ± 0.95, *p* = 0.052)

**Fig. 4:**
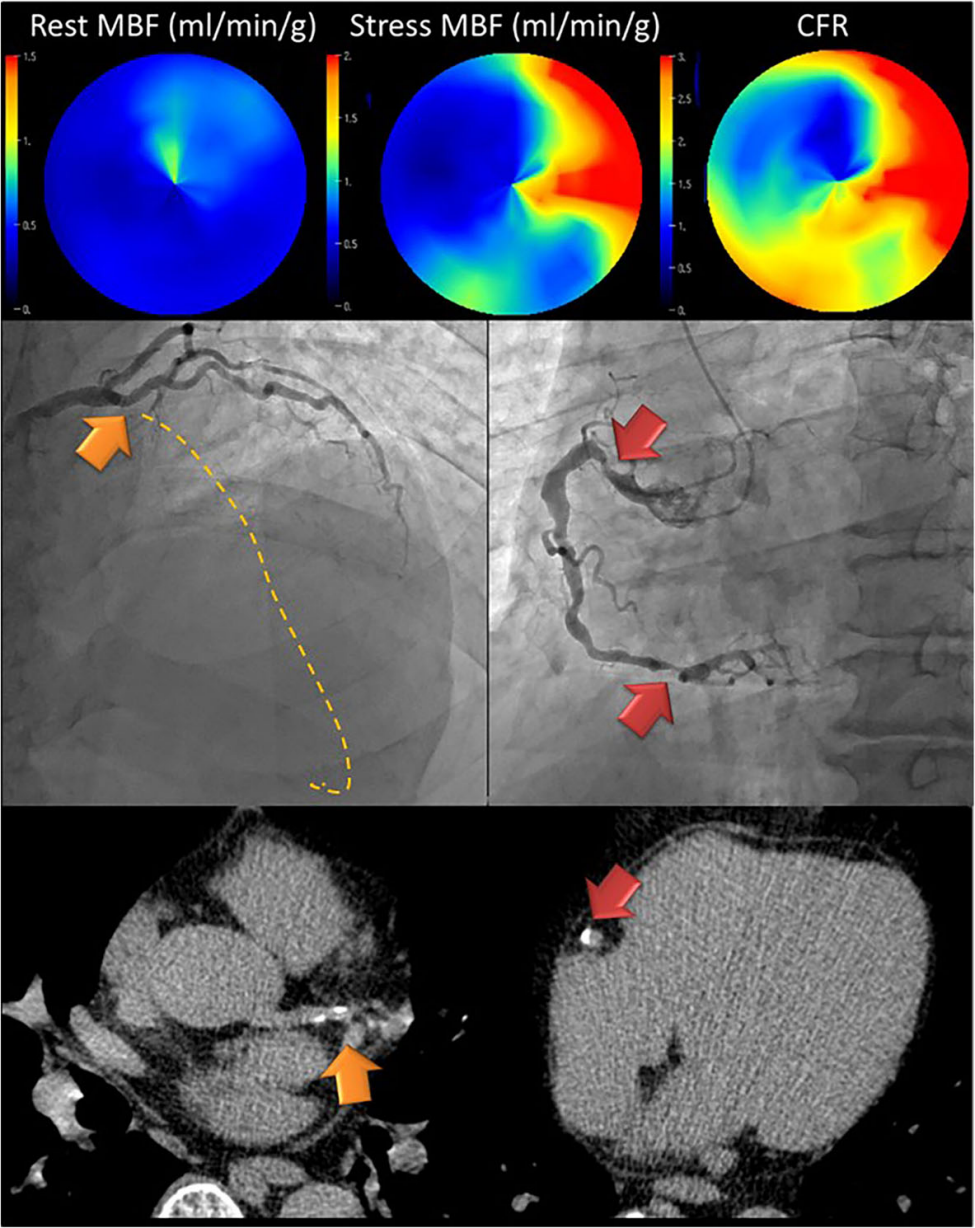
Representative CAD case: A man in his 60s who had significant stenosis in the LAD (#6 100%; *orange arrow*) and RCA (#1 70%, #3 90%; *red arrows*). ^15^O-water PET/CT demonstrated the decrease of stress MBF and CFR in the LAD and RCA territories. The estimated whole CFR was 2.19. The CFRs in the LAD, LCX, and RCA territory were 1.66, 3.11, and 2.01, respectively. Calcifications were seen on the LAD and RCA (Agatston score = 97)

The global CFR of the patients was significantly lower than that of the volunteers (2.75 ± 0.81 vs. 4.54 ± 0.66, *p* = 0.0002; Fig. 2). Similarly, the regional CFR of the patients was significantly lower compared to that of the volunteers (2.63 ± 0.88 vs. 4.53 ± 0.82, *p* < 0.0001). Among the CAD patients, the CFRs in the stenotic regions tended to be lower compared to that in the non-stenotic regions (2.43 ± 0.81 vs. 2.95 ± 0.92, *p* = 0.052) (Fig. 3). The Agatston scores of the patients were significantly higher than those of the volunteers (911 ± 1000 vs. 38 ± 48, *p* = 0.0024). A representative case of a CAD patient is shown in Fig. 4.

## Discussion

We assessed the feasibility of MBF and CFR estimated by ^15^O-water PET/CT with the 3D acquisition mode. A strong inter-observer correlation of MBF and CFR in all subjects was found, and there was little variability of regional MBF and CFR obtained from the healthy control group. The regional MBF and CFR showed a highly homogenous distribution in the healthy volunteers, suggesting that the registration of CT and the dosage of ^15^O-water were appropriate. The CFR in the patients was significantly lower compared to the age-matched control group.

PET is the most reliable method to quantify MBF and CFR, and these quantifications can be applied to the functional severity of coronary stenosis. They can also provide prognostic information about patients with CAD and the coronary risk factors for cardiac events [1, 11]. Supportive evidence has been reported mostly by using a stand-alone PET scanner with two-dimensional (2D) acquisition to quantify MBF and CFR [9, 12]. Roelants et al. directly compared the MBFs between 2D and 3D acquisition modes in dogs with ^15^O-water and ^13^N-ammonia using a stand-alone PET scanner. They concluded that quantification of MBF with 3D acquisition provides results similar to those obtained with the 2D technique, despite a lower activity being injected [13]. In cases of combined PET/CT scans, a misregistration of the PET and CT due to respiratory or cardiac motion and gross physical movement of the patient causes a reduction of the MBF [14, 15].

^15^O-water is known as an ideal PET tracer for the quantification of MBF because of the high extraction fraction. However, ^15^O-water has a limited image quality because it is an inert, freely diffusible tracer [4]. Several automatic and manual methods have been proposed for corrections of the misalignment. Automatic methods were reported; the methods used mostly ^13^N-NH_3_ with high uptake in the myocardium [16–18]. Rajaram et al. reported that the optimal registration of PET and CT was a useful method to avoid this artifact in ^82^Rb PET/CT [6]. As our present findings showed high homogeneity of the regional MBF in the control group, our manual registration method is suitable for ^15^O-water PET/CT.

Tsukamoto et al. reported the values of MBF and CFR obtained from 2D data acquisition using ^15^O-water PET in patients with backgrounds similar to the present study’s patients [19]. In their control group, the estimated MBF at rest, MBF at stress, and CFR were 0.91 ± 0.16 ml/min/g, 3.66 ± 0.81 ml/min/g, and 4.06 ± 0.81, respectively. In the stenotic regions of their CAD group, the estimated MBF at rest, MBF at stress, and CFR were 0.96 ± 0.22 (ml/min/g), 2.19 ± 0.96 (ml/min/g), and 2.35 ± 0.89, respectively. Those data are equivalent to our results from 3D data acquisition.

The quantification of MBF and CFR using PET provides additional diagnostic value for the detection of CAD and can reliably exclude multivessel CAD with very high relative predictive values [20–22]. Our present findings showed that the CFR in stenotic region tended to be lower compared to the non-stenotic region, which is similar to previous reports. The Agatston score and myocardial perfusion imaging are independent predictors. A hybrid PET/CT scanner allows both the integrated assessment of the physiological capacity of blood flow with the CFR and the quantification of the atherosclerotic burden with the Agatston score, which may improve the risk assessment of CAD [23, 24].

A CFR value gives the information of microvascular dysfunction in addition to flow-limiting coronary artery stenosis [1, 11]. Several coronary risk factors such as obesity, diabetes, dyslipidemia, hypertension, renal dysfunction, and smoking are known to adversely affect microvascular function [25–27]. In the present study, the CFR values of the age-matched healthy volunteers were 1.7 times higher than those of the patients even without significant stenosis, which is thought to be related to the high frequencies of hypertension, diabetes mellitus, and smoking history in CAD patients.

This study has some methodological limitations. The sample size was relatively small. However, smaller sample sizes used in previous physiological studies were found to have sufficient power to confirm new methods. The healthy volunteers were not examined via invasive CAG. We also cannot compare 2D and 3D acquisition data because the current PET/CT scanner can obtain data only via 3D acquisition.

## Conclusions

^15^O-water PET/CT with 3D data acquisition can be used reliably for the quantification of functional MBF and CFR in CAD patients.

## Abbreviations

2D: Two-dimensional
3D: Three-dimensional
ATP: Adenosine triphosphate
BMI: Body mass index
CABG: Coronary artery bypass grafting
CAD: Coronary artery disease
CFR: Coronary flow reserve
CT: Computed tomography (CT)
DBP: Diastolic blood pressure
HR: Heart rate
HU: Hounsfield unit
ICC: Intra-class correlation coefficient
LV: Left ventricular
MBF: Myocardial blood flow
PCI: Percutaneous coronary intervention
PET: Positron emission tomography
ROI: Regions of interest
RPP: Rate pressure product
SBP: Systolic blood pressure
